# Impact of Endometriomas and Deep Infiltrating Endometriosis on Pregnancy Outcomes and on First and Second Trimester Markers of Impaired Placentation

**DOI:** 10.3390/medicina55090550

**Published:** 2019-08-30

**Authors:** Carolina Scala, Umberto Leone Roberti Maggiore, Fabio Barra, Matteo Tantari, Simone Ferrero

**Affiliations:** 1 Academic Unit of Obstetrics and Gynecology, IRCCS Ospedale Policlinico San Martino, Largo R. Benzi 10, 16132 Genoa, Italy; 2 Department of Neurosciences, Rehabilitation, Ophthalmology, Genetics, Maternal and Child Health (DiNOGMI), University of Genova, 16126 Genoa, Italy; 3 Piazza della Vittoria 14 S.r.l., 16121 Genova, Italy

**Keywords:** endometriosis, endometrioma, deep endometriosis, adverse pregnancy outcomes, adverse neonatal outcomes, small for gestational age

## Abstract

*Background and objective*: Previous studies did not draw a definitive conclusion about the influence of the role of deep endometriosis (DE) and ovarian endometrioma (OE) as risk factor for developing adverse perinatal outcomes in patients affected by endometriosis. This study aimed to investigate if adverse fetal and maternal outcomes, and in particular the incidence of small for gestational age (SGA) infants, are different in pregnant women with OE versus pregnant women with DE without OE. *Material and methods*: This study was based on a retrospective analysis of a database collected prospectively. The population included in the study was divided into three groups: patients with OE, patients with DE without concomitant OE, and patients without endometriosis (controls). The controls were matched on the basis of age and parity. Demographic data at baseline and pregnancy outcomes were recorded. *Results*: There was no statistically significant difference in first trimester levels of PAPP-A, first and mid-pregnancy trimester mean Uterine Artery Doppler pulsatile index, estimated fetal weight centile, and SGA fetuses’ prevalence for patients with OE, and those with DE without OE in comparison to health women; moreover, there was no statistically significant difference with regard to SGA birth prevalence, prevalence of preeclampsia, and five-minute Apgar score between these three groups. *Conclusions*: The specific presence of OE or DE in pregnant women does not seem to be associated with an increased risk of delivering an SGA infant. These data seem to suggest that patients with endometriosis should be treated in pregnancy as the general population, thus not needing a closer monitoring.

## 1. Introduction

Endometriosis is a chronic estrogen-dependent gynecologic disorder affecting at least 3.6% of women of reproductive age [1]. Pro-inflammatory alterations of both peritoneal cavity and eutopic endometrium have been demonstrated in patients with this hormone-dependent chronic disease, who often suffer from pain symptoms and infertility [2]. Eutopic endometrium and inner myometrium of these women have been demonstrated to have structural and functional abnormalities, not only due to the abnormal expression of genes that are critical for locally producing estrogens and responding to progesterone, but also to alteration of oxidative stress response, presence of inflammatory mediators, cytokines, and various apoptotic markers [3,4,5].

Due to these abnormalities, endometriosis has been associated with defective deep placentation and several obstetrics adverse outcomes [6]. In the literature, several studies reported a correlation between this benign chronic disease and higher risk of spontaneous late abortion [7], preterm premature rupture of the membranes and preterm birth [8,9,10,11,12,13,14], small for gestational age (SGA) [8,9,10,11,12,13,14], pregnancy-induced hypertension [12] and pre-eclampsia [8,11], gestational diabetes [13], and placenta previa [8,9,10,12,14], and other obstetric hemorrhages (such as abruptio placentae and postpartum bleeding) [11,13,14] have been reported for these patients. However, some other studies [15,16] did not definitively confirm the higher risk some of these major obstetric adverse outcomes; thus, this topic remains controversial [17].

Our academic group recently demonstrated that the presence of diffuse adenomyosis in women with endometriosis was more strongly associated with impaired placentation and delivery of SGA infants in comparison to patients with only endometriosis and with focal adenomyosis and concomitant endometriosis [18]; notably, these data may suggest a potential major role of adenomyosis in enhancing the risk of having these obstetrics adverse outcomes.

However, despite this background, the role of each endometriotic phenotypes, in particular ovarian endometrioma (OE) and deep endometriosis (DE), as specific risk factor for developing adverse perinatal outcomes in women with endometriosis, has been not yet investigated. This study aimed to investigate if perinatal and maternal outcomes, particularly with regard to prevalence of SGA infants, are different in pregnant women with OE versus those with DE without OE.

## 2. Materials and Methods

### 2.1. Study Design and Population

This study was done by performing a retrospective analysis of a prospective database collected between January 2017 and June 2018. Women included in the study signed a general consent form for using their clinical data for scientific purposes. The research on humans has been performed by respecting of all the relevant national regulations and institutional policies, in accord to the tenets of the Helsinki Declaration. This study was approved by the Regional Ethic Committee (372REG2017; approval 12 Jan 2018).

Pregnant women with ultrasonographic diagnosis of endometriosis prior to conception were included. The ultrasonographic assessment was performed at any phase of the menstrual cycle regardless of the administration of hormonal treatment (estroprogestins and progestins). Standardized ultrasonographic criteria were employed for the diagnosis of DE [19] and OE [20]; in particular, women with rectosigmoid endometriosis underwent a detailed assessment of intestinal symptoms and a rectal water contrast transvaginal ultrasonography in order to estimate the risk of sub occlusion prior to trying to spontaneously conceive [21,22].

The patients were divided into three groups: women with OE, women with DE without OE, and women without endometriosis (controls). The controls were matched on the basis of age and parity. The controls were selected as the first patient who delivered at our institution, had no prior diagnosis of endometriosis and no symptoms suggestive of this disease (defined as presence of dysmenorrhea, deep dyspareunia, and/or chronic pelvic pain that require analgesic therapy), and had the same range of age of the cases with endometriosis (defined as 18–25, 26–30, 31–35, 35–40, and >41 years old).

Women with previous ultrasonographic diagnosis of uterine adenomyosis [23], with chronic hypertension disease, previous uterine surgery or malformations, and known autoimmune diseases were excluded. Previous surgery for endometriosis was not considered an exclusion criterion for the study if, after surgery, the presence of persistent or recurrent endometriosis was demonstrated at the ultrasonographic assessment. Moreover, pregnancies characterized by major fetal structural abnormalities and/or fetal aneuploidy, obtained by assisted reproductive techniques (ART) and multiple gestations were excluded.

In the first pregnancy-trimester, the measure of crown–rump length (CRL) was used for dating pregnancies according to the NICE (National Institute for Health and Clinical Excellence) guidelines [24]. At the time of 11–14 weeks of pregnancy, PAPP-A levels were measured as first-trimester combined screening test for Down syndrome. Both at the time of routine ultrasonography at 11–14 weeks and of routine anomaly abdominal ultrasonography at 19–23 weeks of pregnancy, uterine artery (UtA) Doppler indices were evaluated; pulsatility index (PI) of the left and the right UtA was averaged to obtain mean PI, which was plotted against a published reference range [25]. During the routine anomaly scan, cervical length was measured by transvaginal ultrasonography following standard parameters: a short cervix was defined if characterized by length ≤25 mm [26]; the suggestion of daily use of vaginal progesterone (200 mg, micronized progesterone capsules) and bed rest were given to women with short cervix in order to prevent preterm birth, according to our institution protocol. During the third pregnancy trimester, at 29–34 weeks, an ultrasonographic scan was done in all the patients to evaluate fetus growth. The administration of aspirin at low-doses as prevention for preeclampsia was not allowed during the study period.

GE Voluson E6 (GE Healthcare, Zipf, Austria) was employed for all the ultrasonographic assessments. At the first study visit, baseline maternal characteristics, including age, ethnic origin, and body mass index (BMI) were recorded. The maternal and neonatal outcomes of each pregnancy were collected. Delivery or follow-up scans were arranged as appropriate for any suboptimal assessments.

Gestational complications were defined with standardized criteria: pregnancy induced hypertension (PIH), detecting after 20 gestation weeks a blood pressure persistently over 140/90 mmHg in a woman with previously normal pressure values; preeclampsia, in case of gestational hypertension and concomitant proteinuria (>300 mg/24 h); preterm birth, indicating a delivery before the completion of 37 gestation weeks; and SGA, in case of an infant with birth weight less than the 10th centile for gestational age.

### 2.2. Statistical Analysis

The Kolmogorov–Smirnov test of normality was used for assessing the distribution of data, which were expressed as mean (SD), or median and interquartile range as appropriate. Categorical variables were described as number (%). The correlations between continuous variables were evaluated by Pearson coefficient or by Spearman rho and those between categorical variables were evaluated by Pearson ×2 test. Continuous variables were compared by Mann–Whitney and independent t-tests. Mean UtA Doppler PI, estimated fetal weight (EFW) centiles, and z-scores were calculated by using appropriate previously described reference ranges [24]. Mean UtA Doppler PI was corrected for gestational age; multiple of medians were calculated by using the reference ranges extracted from the published centiles [24]. Logistic regression analysis was used to evaluate the association between maternal characteristics, first- and second-trimester markers, and fetal outcomes for women with OE and DE without OE; *P* < 0.05 was considered statistically significant. Appropriate statistical software (SPSS 20.0; SPSS Inc, Chicago, IL) was employed for the statistical data analysis.

## 3. Results

Table 1 reports the demographic and pregnancy-related characteristics of women of the study.

There was no statistically significant difference in the baseline data within the three study groups. Overall, 160 pregnant women had complete follow-up, as required for being eligible for the study analysis; within this population, 40 (25%) had OE, 40 (25%) had DE, and 80 (50%) had no endometriosis.

A statistically significant difference was not observed in the first trimester levels of PAPP-A, first trimester and mid-pregnancy mean UtA Doppler PI, EFW centile, and prevalence of SGA fetuses between patients presenting with OE and healthy women; moreover, no statistically significant difference was found in the prevalence of preeclampsia, SGA infants, and five-minute Apgar score between these two groups (Table 1). Moreover, a statistically significant difference was not observed in the first trimester levels of PAPP-A, first trimester and mid-pregnancy mean UtA Doppler PI, EFW centile, and prevalence of SGA infants between patients presenting with DE without OE and healthy women. No statistically significant difference was again found in the prevalence of preeclampsia, SGA infants, and five-minute Apgar score between these two groups (Table 1).

**Figure 1:**
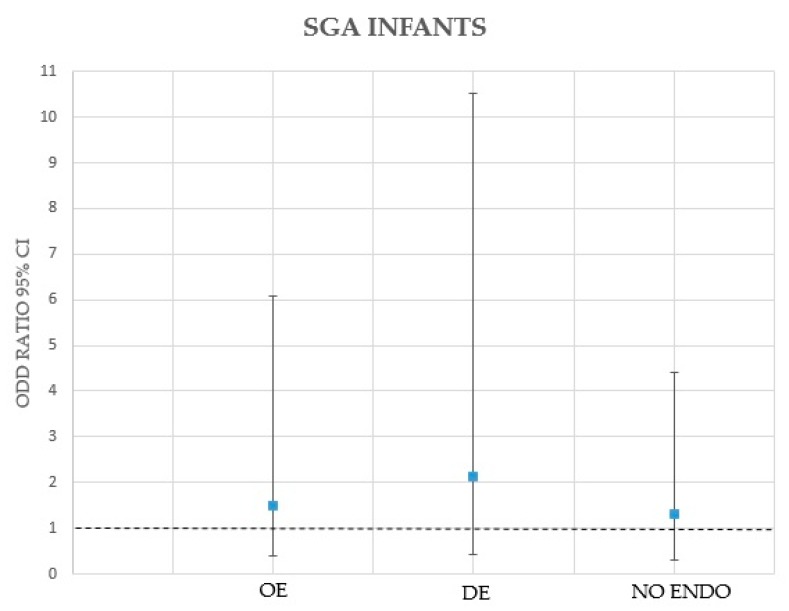
Odds ratios with 95% CIs for delivering SGA infants in women with ovarian endometriomas (OE) and deep endometriosis (DE) in comparison to healthy women (NO ENDO).

The correlation between maternal and pregnancy specific characteristics with SGA and OE and DE was done by logistic regression analysis; Table 2 reports the data related to this analysis. Either the presence of OE nor that of DE without OE were found independently associated with delivering SGA infants (Figure 1).

## 4. Discussion

The results obtained from this study demonstrate that the presence of OE or DE in pregnant women is not associated with an increased risk of delivering SGA infants. During the scan assessment in the 3rd trimester of pregnancy, the prevalence of SGA infants was similar within the three study groups (OE: 8.3%; DE: 7.5%; healthy women: 8.8%); similar results were observed for the prevalence of SGA infants at birth (OE: 8.3%; DE: 7.5%; healthy women: 10.0%). When assessed singularly, conventional risk factors for placental insufficiency, such as BMI, PAPP-A, and mean UtA Doppler PI in first and the second trimesters of pregnancy, did not demonstrate a significant correlation with the presence of OE or DE. More importantly, logistic regression analysis showed that either the presence of OE (1.489; 95 CI % 0.366–6.067; *p* = 0.578) nor the presence of DE (2.121; 95 CI % 0.426–10.564; *p* = 0.381) were associated with the occurrence of SGA infants, after adjusting the results for potential confounding variables (maternal age, ethnicity, BMI, PAPP-A, and mean UtA Doppler PI). Thus, these data seem to not support a potential causative link between these endometriotic phenotypes and impaired placentation and subsequent development of SGA births.

According to the existing literature, the relation between endometriosis and adverse obstetrics outcomes, such as preeclampsia and SGA, is still conflicting [11,27]. Two recent systematic reviews with meta-analysis tried to summarize evidence on this topic [28,29]. In both, subgroup analyses for spontaneous and assisted conception were attempted in order to remove the confounding factor represented by assisted reproduction. In general, women with endometriosis were found to have an increased risk of a range of obstetric and fetal complications, although results for specific adverse maternal and neonatal outcomes tended to differ between these two reviews. Specifically, none of the two [30] reported a pooled increased risk for delivering SGA infants in patients affected by endometriosis; only one [30] observed an increased risk of developing PIH. However, evidence from the analysis of data is limited by the quality and heterogeneity of the studies included: for example, the diagnosis for endometriosis is not uniform; moreover, selection of control groups tends to differ across studies, with some studies evaluating fertile patients, sub fertile patients, or patients affected by male factor infertility as non-endometriotic controls.

A not negligible number of recent studies have found both lower and unchanged risks for these outcomes. Hadfield et al. evaluated 208,879 women with a singleton first birth in the period 2000–2005 in the Australian state of New South Wales in a large population study; among them, 3239 had an earlier diagnosis of endometriosis. No association between the presence of endometriosis and pregnancy-induced hypertension or preeclampsia was reported in this study [30]; notably, stratification for ART did not change the results. In another observational study, Benaglia et al. reported an unchanged risk of hypertensive disorders, preterm birth, gestational diabetes, SGA and large for gestational age newborns, and neonatal problems in women affected by endometriosis [15]. Otherwise, Stephansson et al., in a large cohort of women affected by this chronic benign disease, found an increased risk of pre-eclampsia, preterm birth, antepartum bleeding/placental complications, and cesarean section, but any statistically significant association with SGA infants or stillbirth was not found [14].

Recently, our academic group demonstrated that the presence of diffuse adenomyosis in pregnant women affected by endometriosis is strongly associated with SGA infants, thus suggesting a causative relationship between diffuse adenomyosis and placental dysfunction [18].

The current study investigated, for the first time in the literature, the influence of OE and DE without OE on adverse pregnancy outcomes in women who conceived spontaneously, revealing that neither the presence of OE nor that of DE alone should not be considered relevant risk factors for placental impairment and consequently delivering SGA infants.

This study is characterized by some limitations: firstly, its design is retrospective, although the data were prospectively collected. Furthermore, its sample size is relatively small and this could be considered an impediment for definite conclusions, especially when performing subgroup analysis. However, the study population was highly selected, being composed of women with endometriosis who spontaneously conceived. Given that the main aim of the study was to give information for clinical practice, we were interested in associations of relevant size; in the near future, these preliminary findings may pave the way for trials with larger sample sizes. A further limitation of the current study is that the presence of specific localizations of endometriosis was assessed before conception by ultrasonography. Ideally, a diagnostic laparoscopy before conception would provide a better assessment of the disease but obviously, it is not ethically acceptable to perform a surgical procedure only for this purpose; anyway, because of this study design, we were not able to determinate whether some patients with OE had small DE lesions (main diameter less than 1 cm) not detected by ultrasonography; in contrast, it seems unlikely that OEs were not diagnosed by ultrasonography in the DE group. Similarly, the presence of superficial peritoneal endometriosis in the control group cannot be excluded. However, considering that endometriosis has a low prevalence (about 4%) in the general population [1] and that the control patients did not have pain symptoms requiring antalgic therapy prior to conception, it seems unlikely that a relevant percentage of control women had undiagnosed endometriosis.

A strength of this observational study is that patients with OE and DE without OE were separately studied in subgroups and compared to women without endometriosis; this has been done in order to better understand the impact of each phenotype of endometriosis on specific adverse pregnancy outcomes, and in particular on delivering SGA infants. Moreover, patients who conceived by ART procedures were excluded, thus eliminating the potential bias related to higher prevalence of adverse pregnancy outcomes, such as in cases of preeclampsia.

## 5. Conclusions

The current study shows that the presence of OE and DE without OE are not risk factors of delivering an SGA infant. Thus, patients affected by endometriosis should be treated during pregnancy as the general population, not needing closer monitoring.

## Figures and Tables

**Table 1:** Comparison between pregnant women with ovarian endometriomas and deep endometriosis and women without endometriosis.

	DE (*n* = 40)	OE (*n* = 40)	No Endo (*n* = 80)	*P* value (OE vs no Endo)	*P* value (DE vs no Endo)
**Demographics**					
Maternal age (years, median, IQR)	30.2 (26.8–33)	30.4 (27.75–33)	30.3 (27.0–33.0)	0.933	0.882
Nulliparous (*n*, %)	34 (85.0)	35 (87.5)	69 (86.2)	0.849	0.853
BMI (kg/m^2^, median, IQR)	24.8 (20.4–27.2)	23.8 (21.0–25.3)	25.1 (21.5–26.7)	0.152	0.763
Race (*n*, %)				0.143	0.110
● Caucasian	31 (77.5)	32 (80.0)	73 (91.2)		
● Afro-Caribbean	6 (15.0)	4 (10.0)	5 (6.2)		
● Asian	3 (7.5)	4 (10.0)	2 (2.5)		
● Others	0 (0)	0 (0)	0 (0)		
Previous early miscarriage (*n*, %)	2 (5.0)	2 (5.0)	4 (5.0)	1.000	1.000
Smoking (*n*, %)	5 (12.5)	5 (12.5)	18 (22.5)	0.190	0.190
Surgical/histological diagnosis of disease (*n*, %)	12 (30.0)	11 (27.5)	-	-	
● Rectovaginal (*n*, %)	18 (45.0)	-	-		
● Colorectal (*n*, %)	5 (12.5)	-	-		
● Uterosacral (*n*, %)	23 (57.5)	-	-		
● Bladder (*n*, %)	1 (2.5)	-	-		
**1st and 2nd Trimester Variables**					
PAPP-A (MoM, median, IQR)	1.17 (0.64–1.56)	0.99 (0.58–1.37)	1.09 (0.66–1.55)	0.411	0.502
BhCG (MoM, median, IQR)	1.18 (0.66–1.42)	1.13 (0.56–1.59)	1.01 (0.56–1.36)	0.384	0.205
Mean UtA PI 1st trimester (median, IQR)	1.67 (1.37–1.90)	1.96 (1.33–2.03)	1.64 (1.28–1.98)	0.590	0.806
Mean UtA PI 1st trimester z-scores (mean, SD)	−0.09 (±1.37)	0.03 (±1.59)	−0.15 (±1.58)	0.553	0.850
Mean UtA PI 2nd trimester (median, IQR)	0.94 (0.74–1.12)	0.92 (0.76–1.06)	0.96 (0.75–1.13)	0.591	0.733
Mean UtA PI 2st trimester z-scores (mean, SD)	0.08 (±0.61)	−0.09 (±1.02)	0.09 (±0.87)	0.322	0.561
Short cervix (<25 mm)	0 (0)	1 (2.5)	1 (0.8)	0.614	0.478
**Scan Assessment During the 3rd Trimester of Pregnancy**					
Gestational age 3rd trimester scan (median, IQR)	31.7 (30.5–33.2)	31.8 (30.6–33.2)	31.7 (30.5–33.2)	0.896	0.965
EFW (g, mean, SD)	1868 (±291)	1944 (±284)	1895 (±287)	0.389	0.200
EFW centile (mean, SD)	51.0 (±31.0)	56.6 (±32.6)	53.3 (±31.8)	0.593	0.296
SGA fetuses (*n*, %)	10 (8.3)	3 (7.5)	7 (8.8)	0.815	0.815
**Pregnancy and Perinatal Outcome**					
Gestational age delivery (median, IQR)	39.2 (38.1–40.5)	39.1 (38.0–40.5)	39.0 (38.1–40.5)	0.934	0.806
Birth Weight (mean, SD)	3334 (±495)	3368 (±497)	3337 (±515)	0.754	0.922
Birth weight (centile, mean, SD)	50.0 (±27.9)	52.7 (±28.4)	50.9 (±29.2)	0.744	0.655
SGA (*n*, %)	10 (8.3)	3 (7.5)	8 (10.0)	0.655	0.350
Five-minute Apgar < 7 (*n*, %)	5 (4.1)	2 (5.0)	4 (5.0)	1.000	0.518
Preeclampsia (*n*, %)	9 (7.5)	4 (10.0)	6 (7.5)	0.640	1.000

Data are shown as median (interquartile range) or number (%). Endometriosis: Endo; deep infiltrating endometriosis: DE; ovarian endometrioma: OE; body mass index: BMI; pregnancy-associated plasma protein A: PAPP-A; beta human chorionic gonadotropin: BhCG; estimated fetal weight: EWF; small for gestational age: SGA; uterine artery: UtA; pulsatility index: PI.

**Table 2:** Logistic regression analysis for prediction of SGA.

SGA (*n*)	OR	95% CI	*p*-value
Maternal age	1.038	0.893–1.207	0.628
BMI	0.977	0.868–1.100	0.704
PAPP-A (MoM)	0.842	0.309–2.296	0.737
UtA mean PI (2nd trimester)	0.359	0.036–3.579	0.383
OE	1.489	0.366–6.067	0.578
DE	2.121	0.426–10.564	0.381

Deep infiltrating endometriosis: DE; ovarian endometrioma: OE; body mass index: BMI; beta human chorionic gonadotropin: BhCG; pregnancy-associated plasma protein A: PAPP-A; uterine artery: UtA; pulsatility index: PI; small for gestational age: SGA.
